# Single-cell RNA-seq and machine learning identify HMGN2 as a lactylation-associated hub gene in heart failure

**DOI:** 10.1371/journal.pone.0357971

**Published:** 2026-09-21

**Authors:** Anzhen Xu, Yanbo Li, Qing Gong, Hanrui Sun, Jiayu Shi, Jingyu Li, Pengyang Gu, Jixu Shi, Xiang Li, Qi Lu

**Affiliations:** Department of Cardiology, Affiliated Hospital of Nantong University, Nantong, China; Huazhong Agriculture University, CHINA

## Abstract

Heart failure (HF) is a diverse condition characterized by dysregulated lactate metabolism and histone lactylation, which may contribute to maladaptive cardiac remodeling. However, the cell type-specific mechanisms underlying these processes remain poorly understood. To address this gap, we integrated single-cell RNA sequencing (scRNA-seq) data from human failing and non-failing control hearts and assessed lactylation activity across identified cell types using multiple gene set scoring methods. Uniform manifold approximation and projection (UMAP) analysis revealed that fibroblasts and smooth muscle cells (SMC) exhibited the highest lactylation signature scores, with overall lactylation activity considerably higher in HF compared to controls. Seven potential lactylation-related genes (LRGs) were found using machine learning algorithms, including Boruta, Random Forest, LASSO, and XGBoost. Among these, HMGN2, NUCKS1, and VIM were significantly upregulated in HF, with HMGN2 demonstrating the highest consistency across datasets as a reliable biomarker. Gene set enrichment analysis (GSEA) and transcription factor-miRNA (TF-miRNA) regulatory network analysis connected HMGN2 to immune-inflammatory signaling pathways and protein homeostasis dysregulation. Pseudotime trajectory and intercellular interaction analyses further indicated a progressive rise in lactylation scores and HMGN2 expression along fibroblast and smooth muscle cell differentiation paths, suggesting potential crosstalk with immune cells. In vivo validation using a mouse model of pressure overload-induced HF confirmed cardiac hypertrophy and fibrosis, accompanied by increased HMGN2 expression. These results indicate that lactylation activity is selectively enhanced in HF and identify HMGN2 as a candidate hub gene associated with HF progression. This integrative multi-omics study provides the first cell-type-resolved map of lactylation in HF and highlights HMGN2 as a promising therapeutic target.

## 1. Introduction

HF is a heterogeneous clinical syndrome characterized by impaired ventricular filling or ejection of blood, resulting in the heart’s inability to deliver sufficient cardiac output to meet the body’s metabolic demands or requiring elevated ventricular filling pressures that precipitate systemic circulatory dysfunction. HF signifies the final stage of various cardiovascular illnesses, impacting around 38 million individuals globally. High readmission rates and progressive functional decline significantly compromise patients’ quality of life and clinical outcomes [1,2]. The complexity of HF stems from the interaction of several etiologies and pathological mechanisms, where diverse risk factors such as hypertension, coronary artery disease, and diabetes synergistically compromise cardiac function. Furthermore, symptoms in the initial compensatory phase are often mild and easily misattributed to fatigue or aging, leading to a postponed diagnosis until the irreversible decompensated phase. At the molecular level, HF progression involves hyperactivation of neuroendocrine systems, notably the renin-angiotensin-aldosterone system (RAAS) and the sympathetic nervous system, which exacerbate myocardial stress responses. Also, TGF-β/Smad pathway-mediated fibrotic remodeling impairs ventricular compliance, while metabolic abnormalities such as compromised fatty acid oxidation also play crucial roles. Despite therapeutic advances, a comprehensive knowledge of cell-specific mechanisms driving HF progression remains unclear. At the same time, the slow discovery of stage-specific biomarkers hinders disease trajectory prediction and impedes early precision therapies. Consequently, there is an immediate necessity to employ multi-omics integration approaches to elucidate the cellular environment of HF and pinpoint critical treatment targets.

Lactylation, a recently identified post-translational modification mechanism discovered in 2019, involves the covalent attachment of lactate molecules to the ε-amino groups of lysine residues.Similar to other dynamic PTMs like SUMOylation [3], dynamically altering target protein structures an These findings underscore that lactate not only fuels metabolism but also acts as an epigenetic signal d interaction networks to connect cellular metabolic states with functional adaptation. Mechanistically, lactylation interacts with glycolysis and lactate production pathways, positioning it as a metabolic sensor that integrates energy status with subsequent signaling [4,5]. Research indicates that lactylation influences AMPK-regulated energy balance, histone modification-related epigenetic pathways, and metabolism-immune communication networks [6]. For instance, it remodels immunometabolic microenvironments by regulating checkpoints in macrophages and T cells. Functionally, lactylation governs immune polarization (M1/M2 macrophage conversion), metabolic reprogramming in the tumor microenvironment, regulation of pyroptosis/ferroptosis, and gene transcription via histone H3K18 lactylation. Recent studies have implicated aberrant lactylation in tumor progression, chemoresistance, and fibrotic disorders through the stabilization of oncoproteins or extracellular matrix (ECM) components [7]. However, the role of lactylation in HF remains controversial: some studies suggest cardioprotective effects via inflammation suppression [8], while others implicate pathological lactate accumulation in accelerating myocardial fibrosis [9]. These discrepancies may stem from tissue-specific metabolic milieus and technical limitations in detecting dynamic modifications. Our study pioneers a systematic dissection of the spatiotemporal regulatory network of lactylation in HF models using single-cell transcriptomics, offering fresh insights into its dual roles in cardiac remodeling.

scRNA-seq has revolutionized our understanding of cellular heterogeneity by enabling transcriptomic profiling at single-cell resolution [10]. This technology is crucial for HF research, where microenvironment-specific lactylation—a novel post-translational modification mechanism—may drive disease progression. Despite established links between lactate metabolism and cardiac dysfunction, systematic exploration of lactylation-associated genes in HF at single-cell resolution remains markedly deficient. We established an integrated analytical framework combining scRNA-seq data from HF tissues, bulk transcriptomic analysis of lactylation-associated pathways, and machine learning-driven biomarker discovery [11]. Single-cell analysis revealed distinct cellular subpopulations with lactylation signatures in HF, while cross-cell-type differential expression analysis identified disease-specific lactylation-associated genes. Integrating multi-omics data with machine learning algorithms systematically identified diagnostically valuable lactylation-associated biomarkers. Subsequent functional validation in transverse aortic constriction (TAC)-induced animal models and phenylephrine (PE)-stimulated cardiomyocytes confirmed the biological relevance of candidate targets, guided by bioinformatic predictions. This multi-tiered investigative strategy delineates the dynamic regulatory role of lactylation in HF pathogenesis. Our findings provide new insights into the functioning of lactylation-associated genes in HF and reveal underlying molecular networks, laying critical theoretical groundwork for precision HF treatments.

## 2. Methods

### 2.1 Data acquisition

In this investigation, human heart scRNA-seq data were obtained from the GSE247468 dataset in the Gene Expression Omnibus (GEO) database (https://www.ncbi.nlm.nih.gov/geo/). This dataset comprised samples from 2 healthy donors and 4 HF. The training dataset was generated from GSE57338 in the GEO database, which contained RNA expression patterns of 313 people with and without HF. For validation, three distinct approaches were integrated: (1) Cardiac tissue sequencing from TAC-induced HF mouse models; (2) The GSE42955 dataset, which included 29 human left ventricular samples (12 with dilated cardiomyopathy, 12 with ischemic cardiomyopathy, and 5 controls); (3) The GSE76701 dataset, which comprised 8 human heart specimens (4 from failing hearts and 4 from non-failing donors). Additionally, 332 LRGs curated from previous research [12] were incorporated into the analysis. Although this LRG set was initially identified in a hepatocellular carcinoma (HCC) context, the fundamental biochemical pathway of lactylation—from lactate conversion to lactyl-CoA and subsequent modification by core writers (e.g., EP300) and erasers (e.g., HDACs)—is highly conserved across various eukaryotic cell types and diseases, including cardiovascular pathologies. Due to the current lack of a well-curated, HF-specific lactylome database, this comprehensive gene set was used to evaluate lactylation-associated transcriptomic signatures.

### 2.2 ScRNA-seq dataset analysis

Raw scRNA-seq data were systematically analyzed using the Seurat R package [13]. Following the conversion of raw data into Seurat objects, stringent quality control criteria were applied based on data distribution characteristics and empirical thresholds (nFeature_RNA: 200−5000; nCount_RNA: 200−30,000; mitochondrial gene percentage pMT ≤ 30%; hemoglobin gene percentage pHB ≤ 5%). Low-quality cells were filtered out using the subset function. The retained high-quality cellular data underwent LogNormalize standardization, followed by identification of highly variable genes (HVGs). Principal component analysis (PCA) was employed for data dimensionality reduction, feature integration, and further dimension reduction. Automated cell clustering was achieved using the “FindNeighbors” and “FindClusters” algorithms, and cell type annotation was performed based on well-established marker genes documented in previous studies [14].

### 2.3 Evaluation of inferred lactylation activity

To evaluate the lactylation-related gene set enrichment score at single-cell resolution, this study adapted the methodological framework of SCENIC’s AUCell for transcription factor activity assessment, integrating five well-established computational algorithms. Following standardized protocols, lactylation-related gene set enrichment scores were calculated using four individual methods: AUCell [15], UCell [16], singscore [17], and AddModuleScore [18]. Their sum was defined as a composite score (Score1). Cells were stratified into high- and low-activity groups based on the median lactylation-related gene set enrichment score across the population. Subsequent Spearman correlation analysis identified genes significantly associated with lactylation activity, while differentially expressed genes (DEGs) upregulated in the high-activity group were detected using Seurat’s “FindMarkers” function [19]. The intersecting genes from correlation analysis and DEGs were subjected to downstream functional exploration.

### 2.4 Enrichment analysis

To comprehensively examine biological pathways enriched in high-lactylation activity clusters, multi-level enrichment analysis was performed on filtered candidate gene sets using the Gene Ontology (GO) and Kyoto Encyclopaedia of Genes and Genomes (KEGG) databases. The “clusterProfiler” R package [20] was employed for functional annotation across three ontological domains: Biological Process (BP), Molecular Function (MF), and Cellular Component (CC), while identifying significantly enriched metabolic and signaling pathways (adjusted p-value <0.05). This integrated analytical approach elucidated the core biological functions of candidate genes and delineated key regulatory networks associated with elevated lactylation activity.

### 2.5 Machine learning algorithms for feature gene selection

A multi-model machine learning strategy was employed to systematically identify key regulators associated with lactylation activity for in-depth analysis of pre-filtered genes. Four feature selection algorithms were integrated: eXtreme Gradient Boosting (XGBoost) [21], Boruta algorithm [22], Random Forest [23], and Least Absolute Shrinkage and Selection Operator (LASSO) regression [24]. Gene importance rankings were independently evaluated using each algorithm, and consensus hub genes were identified through stringent cross-algorithm concordance criteria. To elucidate the molecular mechanisms underlying HF, boxplot analysis was conducted to compare expression profiles of these genes between HF and control groups, with statistical significance assessed using a three-tiered annotation system (***: p ≤ 0.001; **: 0.001 < p ≤ 0.01; *: 0.01 < p ≤ 0.05).

### 2.6 Evaluation of feature gene expression and diagnostic potential in validation cohorts

Cross-species validation was conducted using multi-platform datasets to validate the expression patterns and diagnostic potential of the optimal feature genes in HF tissues. For human cardiac samples, transcriptomic data from three independent HF cohorts (GSE57338, GSE42955, GSE76701) were analyzed via the Wilcoxon rank-sum test to assess expression differences of optimal feature genes between HF and control groups. Additionally, cardiac sequencing data from TAC-induced murine HF models were included for cross-species validation. Receiver Operating Characteristic (ROC) curves were generated across these four datasets to evaluate diagnostic ability, with Area Under the Curve (AUC) values measuring predictive efficacy (AUC > 0.7 indicating strong diagnostic capacity) [25].

### 2.7 GSEA and TF-miRNA

To elucidate the regulatory mechanisms of HMGN2, VIM, and NUCKS1 in HF, TF-miRNA interaction networks were predicted using the KnockTF database and the MultiMiR package [26]. Single-gene GSEA enrichment analysis was performed on HMGN2 through Reactome and KEGG databases [27].

### 2.8 Trajectory analysis

Cells were dichotomously classified to construct cellular differentiation trajectories based on the presence/absence of optimal feature gene expression. The scaled unique molecular identifier (UMI) count matrix extracted from Seurat subsets was used as input for trajectory inference using the Monocle 2 algorithm with default parameters [28].

### 2.9 Interactions between intercellular communication

CellChat [29] was employed to analyze intercellular communication dynamics, using the default CellChatDB ligand-receptor database. Cell type-specific interaction modules were identified by systematically detecting overexpressed ligands or receptors in cellular subsets and evaluating their ligand-receptor interaction strengths.

### 2.10 TAC-induced HF model

This study utilized TAC in 8–10-week-old male C57BL/6 mice (Nantong University Laboratory Animal Center) to minimize the cardiovascular protective effects of estrogen and hormonal fluctuations, randomly assigned to sham or TAC groups. Mice were anesthetized with ketamine (100 mg/kg) and xylazine (2.5 mg/kg) via intraperitoneal injection. A left anterolateral thoracotomy was performed to expose the aortic arch under visual guidance. A 27-gauge needle-guided 6–0 silk suture was ligated around the aortic arch to create TAC, followed by needle withdrawal and wound closure with 4–0 sutures. Sham controls underwent identical surgical procedures without aortic ligation [30]. Postoperative mice were maintained on a temperature-controlled heating pad until full recovery before returning to standard housing. Terminal euthanasia was performed via cervical dislocation under deep anesthesia. All animal experiments were strictly performed in accordance with the guidelines for the care and use of laboratory animals and were approved by the Institutional Animal Care and Use Committee (IACUC) of Nantong University (Approval No. P20251021-005).

### 2.11 RNA-seq sequencing

For RNA-seq analysis of TAC-modeled mice, myocardial tissues from control and 8-week post-TAC groups were dissected and snap-frozen in liquid nitrogen. Total RNA was isolated using TRIzol reagent (Invitrogen) following the manufacturer’s protocols, with strict adherence to RNA integrity and purity standards. RNA concentration and purity (OD260/280 and OD260/230 ratios) were quantified via NanoDrop spectrophotometry, and integrity was verified by 1.5% agarose gel electrophoresis. Ribosomal RNA was depleted using the Ribo-Zero Gold rRNA Removal Kit (Illumina) to enrich mRNA. First-strand cDNA synthesis used random hexamer primers, followed by second-strand synthesis with buffer, dNTPs, RNase H, and DNA Polymerase I. Double-stranded cDNA was purified via magnetic bead-based systems and eluted in EB buffer. Purified cDNA underwent end repair, A-tailing, and Illumina adapter ligation. UNG enzyme treatment degraded uracil-containing second strands. Size selection (200–500 bp) via gel electrophoresis preceded PCR amplification and sequencing on an Illumina Novaseq 6000. The entire experimental workflow was rigorously conducted under stringent quality control measures to ensure high-quality sequencing data with demonstrated reliability.

### 2.12 Evaluation of lactylation activity in HF model

Following the establishment of a PE-induced HF model, H9C2 cardiomyocytes were harvested for subsequent analysis. Total RNA was extracted from cell pellets using TRIzol reagent according to the manufacturer’s protocol, with RNA concentration and purity verified spectrophotometrically. cDNA was synthesized from 1 μg of total RNA using a PrimeScript RT reagent kit with gDNA Eraser. After sealing the plate with the prepared samples, amplification was performed on a real-time PCR detection system. The standard cycling conditions included an initial denaturation at 95°C for 30 s, followed by 40 cycles of denaturation at 95°C for 5 s and annealing/extension at 60°C for 30 s. First, the mRNA expression levels of established cardiac hypertrophy markers atrial natriuretic peptide (ANP), brain natriuretic peptide (BNP), and β-myosin heavy chain (β-MHC) were quantified to validate successful model establishment. The model group exhibited significantly elevated expression of these markers, confirming successful induction of the HF phenotype. Subsequently, the expression level of lactate dehydrogenase A (LDHA) [31], a key enzyme regulating lactate production and lactylation modification, was assessed. Using TB Green Premix Ex Taq II, all reactions were performed in triplicate. The relative expression of target genes, normalized to GAPDH, was analyzed using the 2 − ΔΔCt method.

The primer sequences used were as follows:

LDHA forward, 5’- TAATGAAGGACTTGGCGGATGA-3’ and reverse, 5’- ACCAGCTTGGAGTTCGCAGTTA-3’;
GAPDH forward, 5’- CCTCGTCCCGTAGACAAAATG-3’ and reverse, 5’- TGAGGTCAATGAAGGGGTCGT-3’.
β-MHC forward, 5’- CCCAGAAACAAGTGAAGAGCCT-3’ and reverse, 5’- GTTCCACGATGGCGATGTTC-3’.
ANP forward, 5’- CTTCTTCCTCGTCTTGGCCTTT-3’ and reverse, 5’- TCCAGGTGGTCTAGCAGGTTCT-3’.
BNP forward, 5’- CTTTATCTGTCACCGCTGGGAG-3’ and reverse, 5’- ACAACTTCAGTGCGTTACAGCC-3’.

### 2.13 Echocardiography

Mice were anesthetized with 2% isoflurane. Chest hair was depilated, and ultrasound coupling gel was applied to the anterior thoracic region. Cardiac parameters (IVSd, LVIDd, LVPWd, IVSs, LVIDs, LVPWs, ejection fraction (EF), and fractional shortening (FS)) were acquired in the parasternal long-axis view using a Vevo 2100 high-resolution imaging system equipped with an FMS-250 probe (13–24 MHz), with values averaged over 4–5 consecutive cardiac cycles [32].

### 2.14 Immunostaining and histological analysis

For comprehensive histological analysis of cardiac tissues, samples were prepared using both paraffin-embedded and frozen-section methodologies. Sections of 5 μm thickness obtained from paraffin-embedded tissues were stained with hematoxylin and eosin (H&E) to assess general morphology and with Masson’s trichrome to evaluate collagen content and fibrosis. To specifically examine cardiomyocyte hypertrophy, frozen sections were permeabilized with 0.1% Triton X-100, blocked in 5% bovine serum albumin (BSA), and incubated with FITC-conjugated wheat germ agglutinin (WGA) (1:200) for 30 minutes at 37 °C. Imaging was performed using an epifluorescence microscope.

### 2.15 Western blotting

Protein lysates were prepared from both cardiac tissues of TAC-induced mice and PE-stimulated H9c2 cardiomyocytes. For the *in vitro* model, H9c2 cells were cultured for 48 h, synchronized by serum starvation for 24 h in high-glucose basal medium supplemented with 0.1% 5-bromodeoxyuridine (Brdu) (0.1 mmol/L), and subsequently treated for 48 h as follows: The control group received the synchronization medium (100 µl). The model group was treated with the same medium containing 100 µM PE. Proteins were separated by SDS-PAGE, transferred to a membrane, and probed with specific primary antibodies. To ensure equal loading, membranes were incubated with an anti-α-tubulin antibody (Servicebio, China). The band intensity of the protein of interest, HMGN2 (ABCAM, UK), was quantified using image analysis software and normalized to α-tubulin. The results are expressed as the relative integrated density value compared to the control group. All primary antibody incubations were performed overnight at 4°C [33].

### 2.16 Statistical analysis

Statistical analyses were performed using R 4.1.3 (R Foundation) and GraphPad Prism 9.0 (GraphPad Software, USA). Continuous data are expressed as mean ± standard error of the mean (SEM). Group differences for continuous variables were evaluated with either the Wilcoxon test or *t*-test for two-group comparisons, while one-way ANOVA was applied for multi-group analyses. Intervariable associations were examined via Spearman’s correlation coefficients. All significance thresholds used two-tailed tests with *p* < 0.05 denoting statistical significance. For all in vitro and in vivo experiments, data are presented as the mean ± standard error of the mean (SEM) of independent biological replicates. Specifically, n = 5 per group for bulk RNA-seq; n = 3 per group for echocardiography, histological staining, qPCR, and Western blotting experiments.

### 2.17 Ethical statement

All animal experiments were strictly performed in accordance with the guidelines for the care and use of laboratory animals and were approved by the Institutional Animal Care and Use Committee (IACUC) of Nantong University (Approval No. P20251021-005).

## 3. Results

### 3.1 Schematic of the experimental workflow

**Fig 1:**
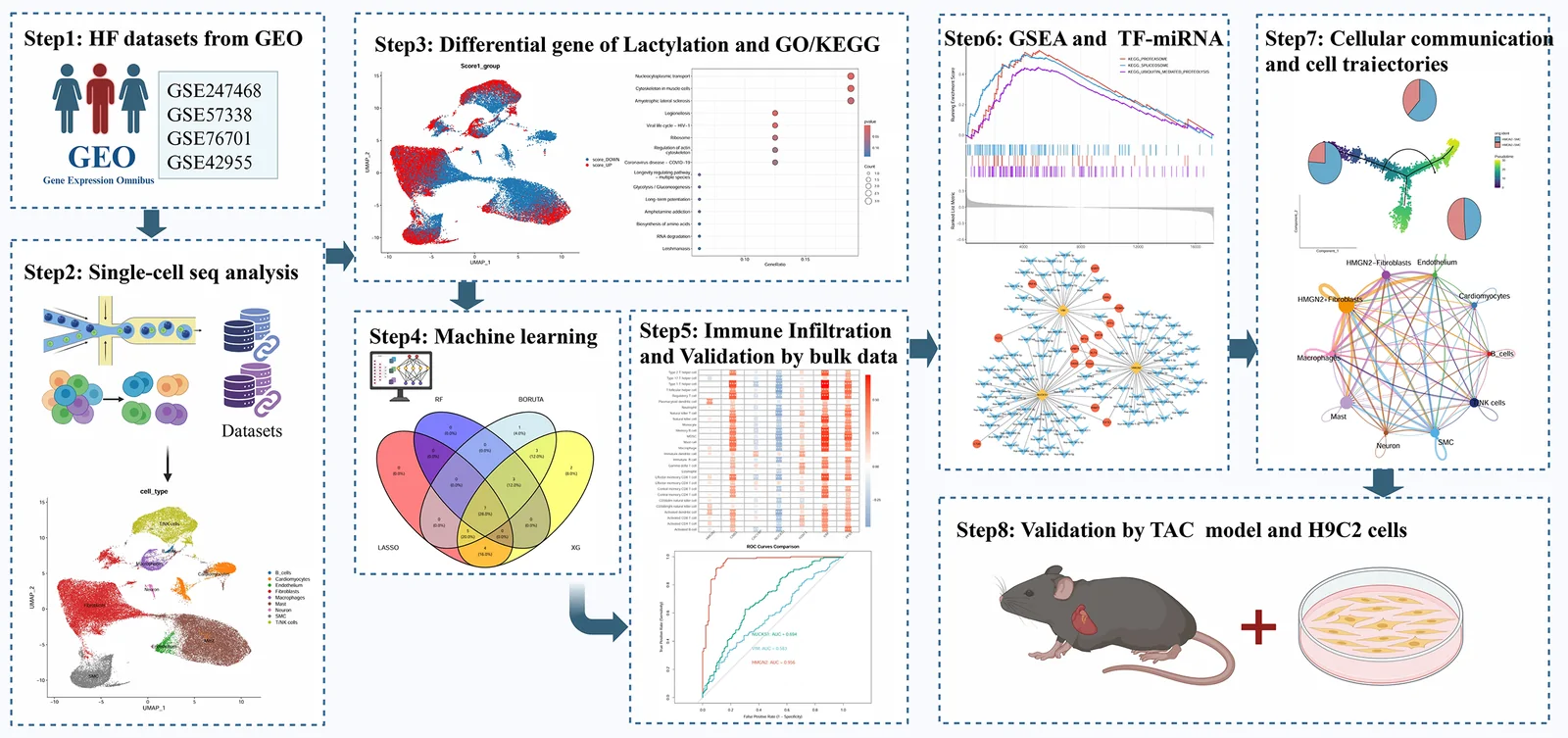
Schematic of the experimental workflow.

A workflow diagram for this study is shown in Fig 1.

### 3.2 ScRNA-seq uncovers cellular heterogeneity and dynamic remodeling in HF

**Fig 2:**
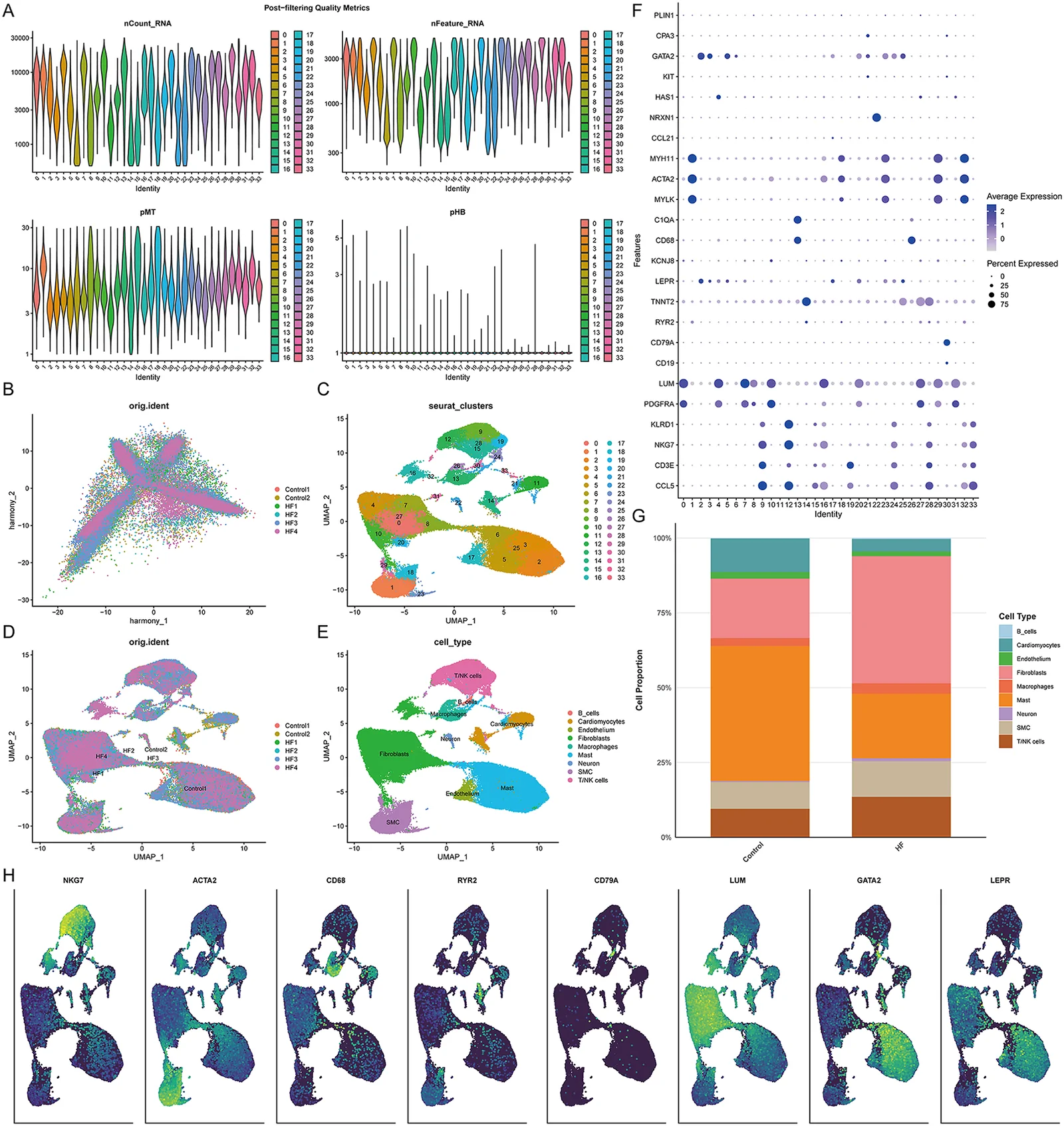
HF Cellular Dynamics Uncovered by scRNA-seq. A. Violin plots of quality metrics (nCount_RNA, nFeature_RNA, pMT, pHB) used to filter cells at empirical thresholds. B. PCA projections before and after Harmony integration demonstrate effective batch-effect correction. C. Seurat-based clustering identifies 34 cell clusters. D. UMAP projection visualizes cluster relationships and sample-specific distributions. E. Annotation using canonical markers defines 9 cell types, including fibroblasts and cardiomyocytes. F. Dot plot validates marker gene expression and statistical significance. G. Stacked bar plot shows proportional shifts: fibroblast expansion and cardiomyocyte loss in HF. H. UMAP heatmap displays marker gene expression with a color gradient.

To evaluate the quality of scRNA-seq data and select high-quality cells for HF analysis, mitochondrial (pMT) and hemoglobin (pHB) gene percentages were calculated using the PercentageFeatureSet function. Then, four quality metrics (nCount_RNA [RNA counts], nFeature_RNA [gene features], pMT, and pHB) were visualized through VlnPlot-generated violin plots. Cell filtering was performed using the subset function with empirically determined thresholds: nFeature_RNA (200–5000), nCount_RNA (200–30000), pMT ≤ 30%, and pHB ≤ 5%, based on data distribution patterns (Fig 2A). Data normalization was performed using LogNormalize with a scale factor of 10,000. 2,000 HVGs were selected for scaling, and PCA was carried out for preliminary data exploration. Initial PCA revealed significant batch effects across sample groups. The integration of the Harmony algorithm, with orig.ident designated as the batch variable, effectively removed technical variations, as evidenced by the post-correction two-dimensional (2D) embeddings (harmony_1/harmony_2 coordinates) that confirmed effective batch effect mitigation (Fig 2B). To investigate the cellular heterogeneity and sample distribution patterns associated with HF, cell clustering was conducted using the Seurat algorithm, resulting in the identification of 34 distinct cell clusters (numbered 0–33) (Fig 2C). Subsequent UMAP visualization provided a clear depiction of both cluster relationships and cellular distributions across different samples (HF1–4, Control1–2) (Fig 2D). Through manual annotation based on canonical marker genes, nine cell types were identified: B cells, cardiomyocytes, endothelial cells, fibroblasts, macrophages, mast cells, neurons, SMC, and T/NK cells (Fig 2E). The accuracy of this annotation was further substantiated by dot plot visualization of marker genes, which showcased both the statistical significance and expression levels of cell type-specific genes (Fig 2F). To investigate the dynamic changes in cellular composition during HF progression, stacked bar plots were generated to compare cell type proportions between the Control and HF groups via scRNA-seq analysis. The analytical results demonstrated a notable expansion of fibroblasts (pink-shaded segment) and a depletion of cardiomyocytes (teal-colored segment) in the HF cohort compared to the Control cohort, with macrophages and mast cells showing distinct redistribution patterns (Fig 2G). Furthermore, gene expression visualization experiments were carried out using single-cell transcriptomic data to analyze the subpopulation-specific expression patterns of marker genes. Cellular distributions were visualized through UMAP dimensionality reduction, and representative marker genes for each major cell type were displayed in heatmaps with a blue (low)-to green/yellow (high) expression gradient (Fig 2H).

### 3.3 Analyzing cell-type-specific lactylation signatures and their functional implications in HF by scRNA-seq

**Fig 3:**
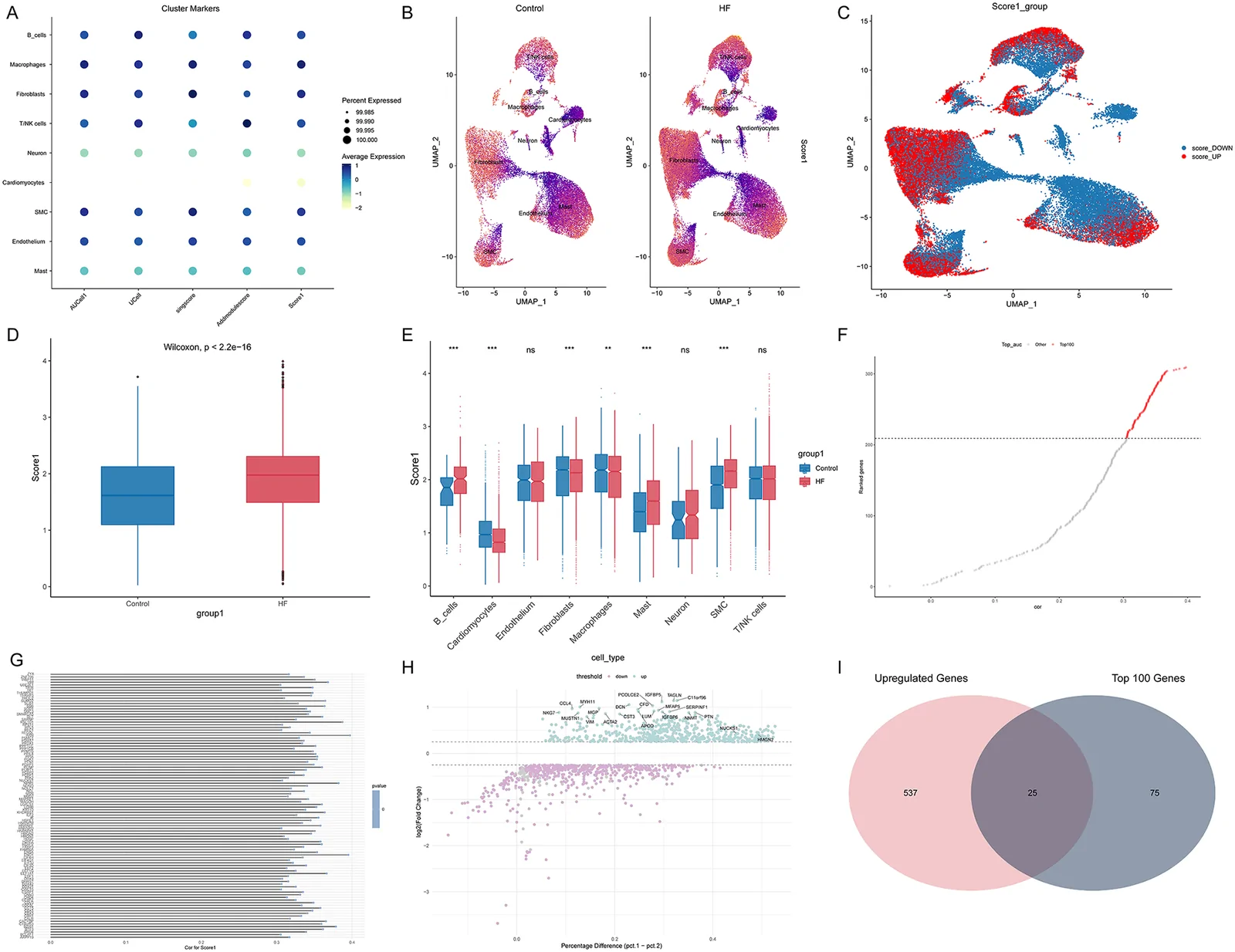
Cell-specific lactylation in HF. A. Elevated lactylation scores in SMCs, fibroblasts, and T/NK cells link them to HF progression. B. UMAP visualization identifies fibroblasts as a primary cellular hub of inferred lactylation activity in HF. C. Fibroblasts and SMCs constitute the cluster with high lactylation-related gene signature scores. D-E. Global elevation of lactylation in HF (p < 2.2e-16), specifically in SMCs, fibroblasts, and immune cells. F-G. Spearman correlation identifies 100 lactylation-associated genes. H. 562 genes are upregulated in high lactylation cells. I. 25 genes overlap between lactylation-associated and HF-upregulated sets.

To decipher the lactylation-associated gene expression patterns across different cardiac cell types and their pathological relevance to HF, we conducted a systematic evaluation of nine cell populations (including B cells, macrophages, and fibroblasts) using five computational algorithms (AUCell, UCell, singscore, Addmodulescore, and Score1 [summation of first four]) based on single-cell transcriptomic data. The results indicated elevated lactylation signature scores in SMCs, fibroblasts, and T/NK cells, suggesting their involvement in HF progression through lactylation pathways. In contrast, neurons showed limited association, providing novel evidence for cell-type-specific metabolic regulation in HF (Fig 3A). To investigate the distributional differences in lactylation-related gene set enrichment scores between the HF and Control groups, we visualized single-cell transcriptomes using UMAP-based dimensionality reduction. This visualization confirmed elevated lactylation activity in fibroblasts as a key pathological feature (Fig 3B). Using predefined lactylation-associated gene sets, we conducted pathway activity scoring (Score1) on single-cell data, followed by UMAP dimensionality reduction for cellular distribution visualization. The results demonstrated that the cluster with high lactylation activity was predominantly composed of fibroblasts and SMCs (Fig 3C). To decipher the disparities in lactylation-associated pathways across cell types during HF, we compared the Score1 lactylation signature scores between the HF and Control groups using the Wilcoxon rank-sum test, both globally and across individual cell types. The results demonstrated a significantly elevated median Score1 in HF versus Control (p < 2.2e-16). These findings indicate that lactylation activity is selectively enhanced in HF, particularly in SMCs, fibroblasts, and immune-related cells (B and mast cells). This suggests that lactylation may contribute to the pathogenesis of HF by modulating vascular remodeling, fibrosis, and inflammatory responses (Fig 3D and 3E). To decipher the regulatory features of lactylation pathway-associated genes in HF single-cell data, we conducted Spearman correlation analysis to quantify associations between gene expression and pathway scores (Score1), followed by functional classification based on correlation coefficients (cor) and gene ranking. Correlation analysis identified 100 genes significantly associated with lactylation modifications (Fig 3F and 3G). Screening of DEGs in HF single-cell data based on lactylation pathway scores revealed 562 upregulated genes between high- and low-expression groups (Fig 3H). Venn diagram analysis was used to assess the overlap between the upregulated gene set (562 genes) and the lactylation-related pathway-associated gene set (Top 100, ranked by correlation). The analysis revealed that 25 genes were common to both sets (Fig 3I). To investigate the biological functions of HF-associated lactylation genes, GO and KEGG enrichment analyses were performed on the 25 overlapping genes. KEGG analysis significantly enriched these genes in nucleocytoplasmic transport, cytoskeleton organization in muscle cells, and amyotrophic lateral sclerosis pathways (S1 Fig). GO analysis revealed that CCs were substantially correlated with focal adhesion, MFs were associated with cadherin binding, and BPs primarily involved ribosome biogenesis and ribosomal subunit export (S1 Fig).

### 3.4 Machine learning-based identification of core feature genes for HF

**Fig 4:**
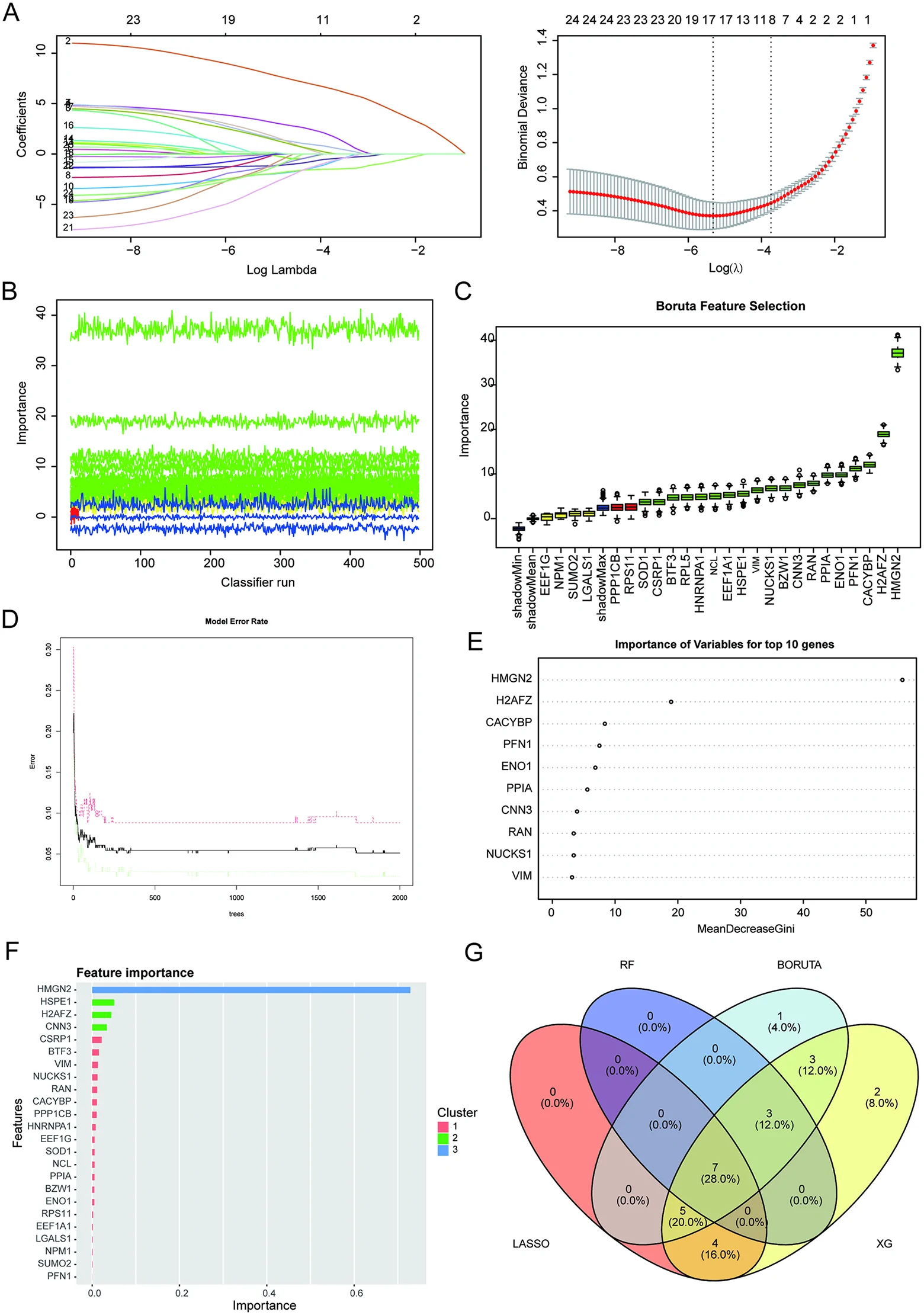
Machine learning identification of core feature genes. A. LASSO regression selected 16 key genes. B-C. The Boruta algorithm identified 19 critical genes. D-E. Random forest prioritized 10 significant genes. F. XGBoost detected 24 candidate genes. G. Integration of all methods revealed 7 core genes (HMGN2, CNN3, CACYBP, NUCKS1, H2AFZ, VIM, PFN1).

Four machine learning algorithms were used to identify key feature genes among the 25 candidate genes. LASSO regression identified 16 key genes with the lowest binomial deviance (Fig 4A). The Boruta algorithm identified 19 critical genes after 500 iterations (Fig 4B and 4C). The random forest algorithm prioritized 10 significant genes (Fig 4D and 4E). XGBoost analysis detected 24 candidate genes (Fig 4F). Seven core feature genes (HMGN2, CNN3, CACYBP, NUCKS1, H2AFZ, VIM, and PFN1) were identified by combining the results of all four algorithms (Fig 4G).

### 3.5 HMGN2 as an optimal feature gene revealed by immune interactions and cross-species validation

**Fig 5:**
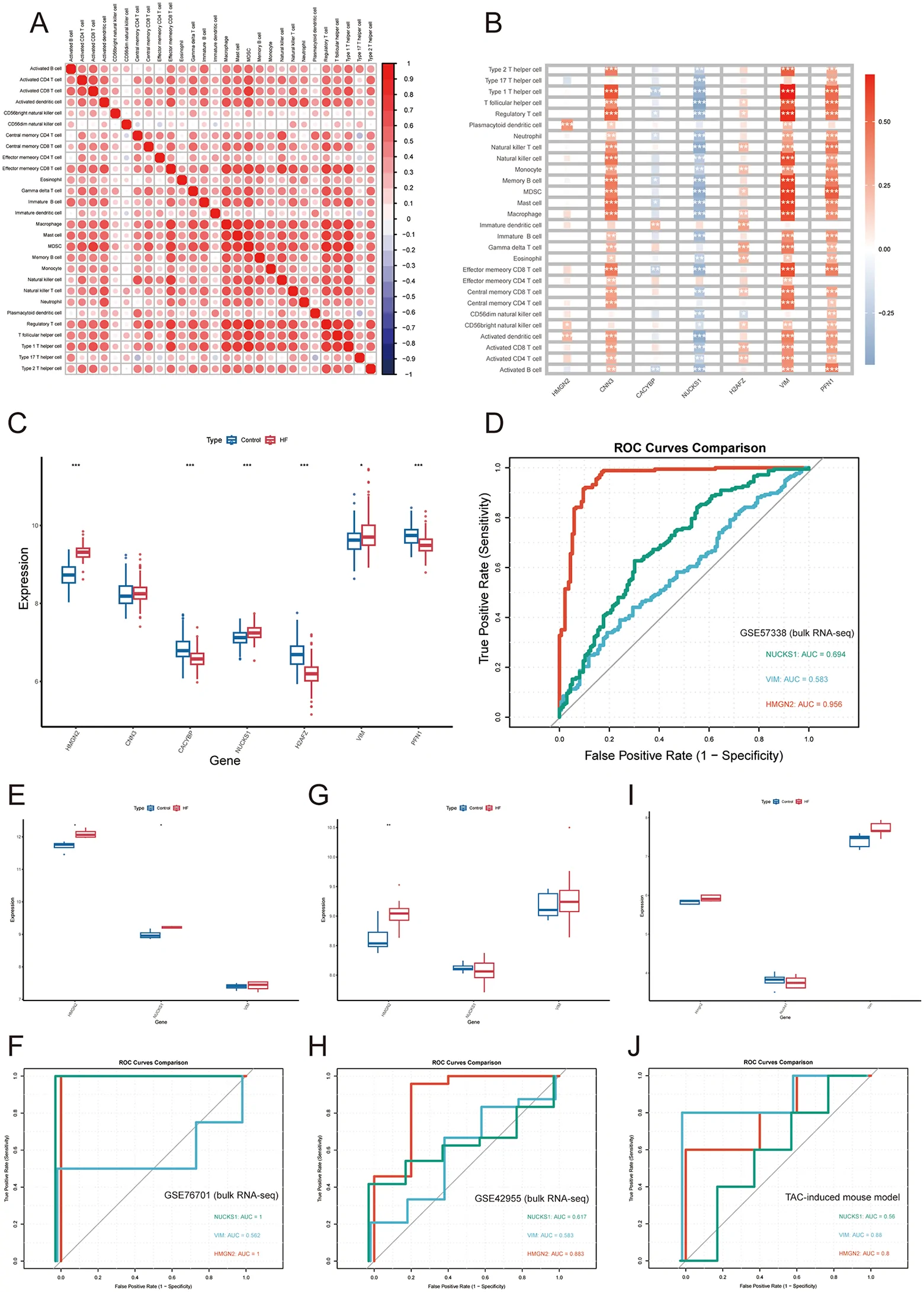
HMGN2 as a conserved HF diagnostic indicator. A-B. All 7 key genes show significant positive correlations with immune cell infiltration (p ≤ 0.001). HMGN2 correlates strongly with dendritic cells. C. HMGN2, NUCKS1, and VIM are upregulated in HF versus controls. D. In cohort GSE57338 (bulk RNA-seq), HMGN2 achieves an AUC of 0.956 for HF diagnosis. E-F. In validation set GSE76701 (bulk RNA-seq), HMGN2 (AUC = 1.0) and NUCKS1 (AUC = 1.0) are upregulated in HF. G-H. In cohort GSE42955 (bulk RNA-seq), HMGN2 remains upregulated (AUC = 0.883). I. In a TAC-induced mouse HF model, HMGN2 (AUC = 0.8) and VIM (AUC = 0.88) show diagnostic value.. J. HMGN2 is prioritized as the core HF gene based on consistent upregulation and cross-species diagnostic performance.

To explore the crosstalk between key genes and the immune microenvironment, ssGSEA-based immune infiltration analysis was performed. The correlations between seven key genes (HMGN2, CNN3, CACYBP, NUCKS1, H2AFZ, VIM, PFN1) and 28 immune cell types were visualized using bubble plots and heatmaps. The bubble plot revealed significant positive correlations between all seven genes and specific immune subclusters. The heatmap further confirmed the statistical significance of these associations, with all marked as *** (p ≤ 0.001). For instance, HMGN2 showed the strongest correlation with plasmacytoid dendritic cells and activated dendritic cells, while CNN3 was highly associated with Type 1 T helper cells. VIM presented a strong correlation with the majority of immune cells (Fig 5A and 5B). To investigate the molecular mechanisms underlying HF and identify potential therapeutic targets, we compared the expression levels of the seven key genes between HF and control groups using box plot analysis, with statistical significance assessed using significance markers. The results showed significant upregulation of HMGN2, NUCKS1, and VIM in the HF group. Based on their expression levels and functional relevance, these three genes were prioritized as optimal hub genes, with subsequent analyses focusing on their regulatory networks and disease associations (Fig 5C). To validate these findings, we evaluated the three candidate biomarkers at the bulk RNA-seq level. In the GSE57338 training cohort, HMGN2 achieved an AUC of 0.956 in HF patients, demonstrating exceptional sensitivity and specificity for distinguishing HF from controls. In contrast, NUCKS1 (AUC = 0.694) and VIM (AUC = 0.583) showed lower diagnostic value (Fig 5D). In the independent validation set GSE76701, differential expression analysis (box plots) and ROC curve analysis confirmed significant upregulation of HMGN2 in HF (AUC = 1.0), consistent with previous findings. NUCKS1 showed marginal upregulation (AUC = 1.0), while VIM displayed no statistical significance (AUC = 0.562) (Fig 5E and 5F). Analysis of the GSE42955 validation cohort using box plots and ROC curves indicated upregulation of HMGN2 (AUC = 0.883), whereas NUCKS1 (AUC = 0.617) and VIM (AUC = 0.583) performed near random chance levels (Fig 5G and 5H). We established a TAC-induced murine model to identify cross-species conserved core genes in HF with clinical translatability and performed RNA sequencing on cardiac tissues for final validation. In murine HF models, HMGN2 and VIM showed AUCs of 0.8 and 0.88, respectively, while NUCKS1 achieved an AUC of 0.56 (Fig 5I and 5J). HMGN2 was ultimately prioritized as the core gene based on its consistent upregulation across datasets, cross-dataset stability in human cohorts, and conserved diagnostic performance in cross-species validations.

### 3.6 Results of single-gene GSEA and TF-miRNA network

**Fig 6:**
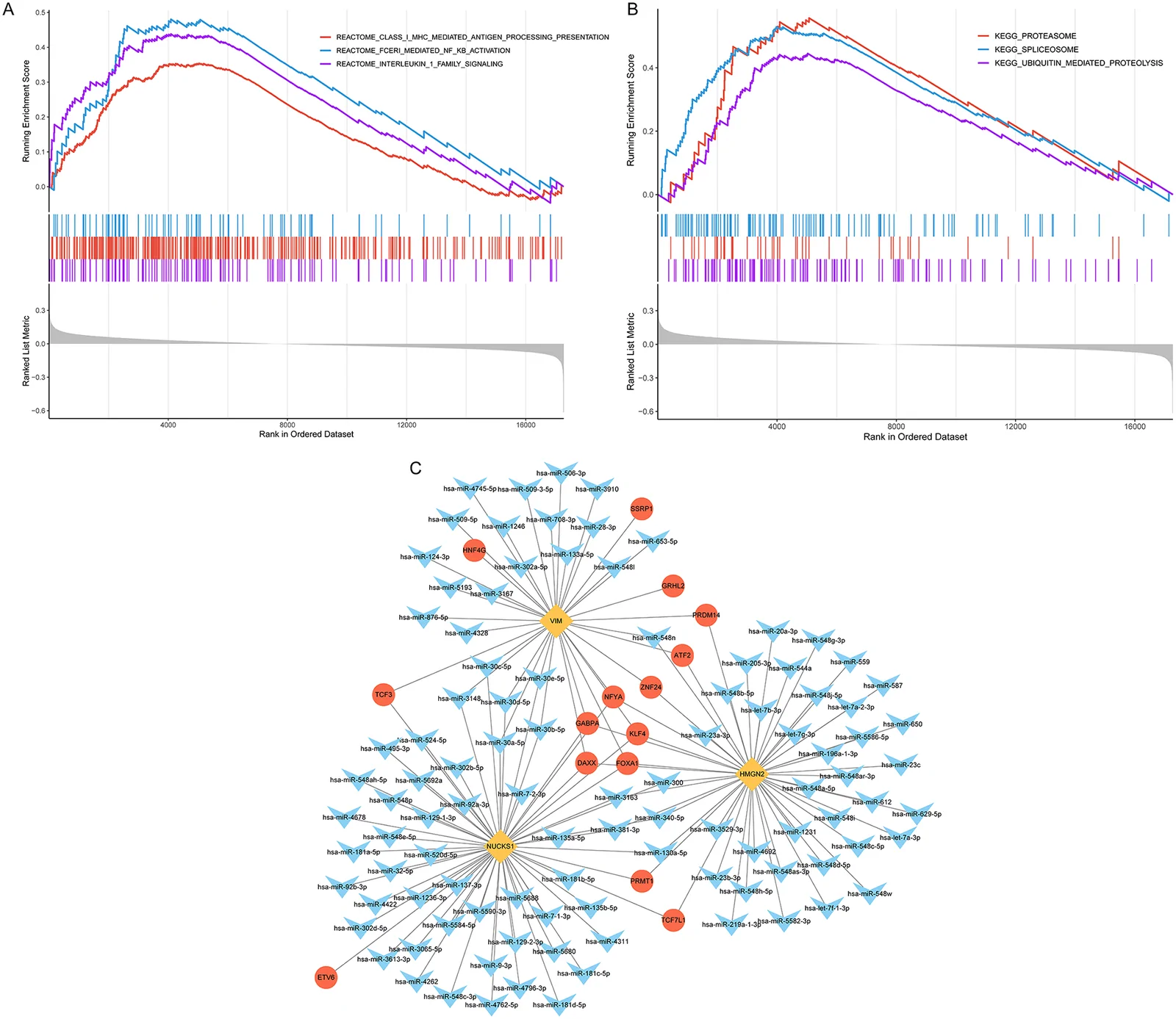
HMGN2-mediated regulation of immune and proteostatic pathways in HF via GSEA and TF-miRNA networks. (A–B) HMGN2-high samples enrich immune (MHC I, NF-κB, IL-1) and protein degradation pathways (proteasome, spliceosome, ubiquitin). (C) TF-miRNA network depicts HMGN2, VIM, and NUCKS1 interactions with TFs and miRNAs.

To systematically investigate the multi-dimensional regulatory mechanisms of HMGN2 as a core gene in HF, we conducted single-gene GSEA using the Reactome and KEGG databases. GSEA revealed significant enrichment of HMGN2-high samples in Reactome pathways, including MHC class I antigen presentation, FcεRI-mediated NF-KB activation, and IL-1 family signaling. These findings suggest that HMGN2 may be involved in HF progression through immune regulation and pro-inflammatory responses. Additionally, KEGG analysis demonstrated prominent enrichment of proteasome, spliceosome, and ubiquitin-mediated protein degradation pathways, indicating that HMGN2 contributes to HF progression through dysregulation of protein homeostasis and defects in transcriptional control (Fig 6A and 6B). These findings suggest that HMGN2 serves as a potential regulatory hub in HF pathology, potentially operating through dual mechanisms: modulation of immune-inflammatory signaling and disruption of protein metabolic homeostasis. To dissect the regulatory mechanisms of HMGN2, VIM, and NUCKS1 in HF, we predicted and constructed their interaction networks with transcription factors (TFs) and miRNAs using KnockTF and MultiMiR (Fig 6C).

### 3.7 Results of trajectory analysis and cell interactions

**Fig 7:**
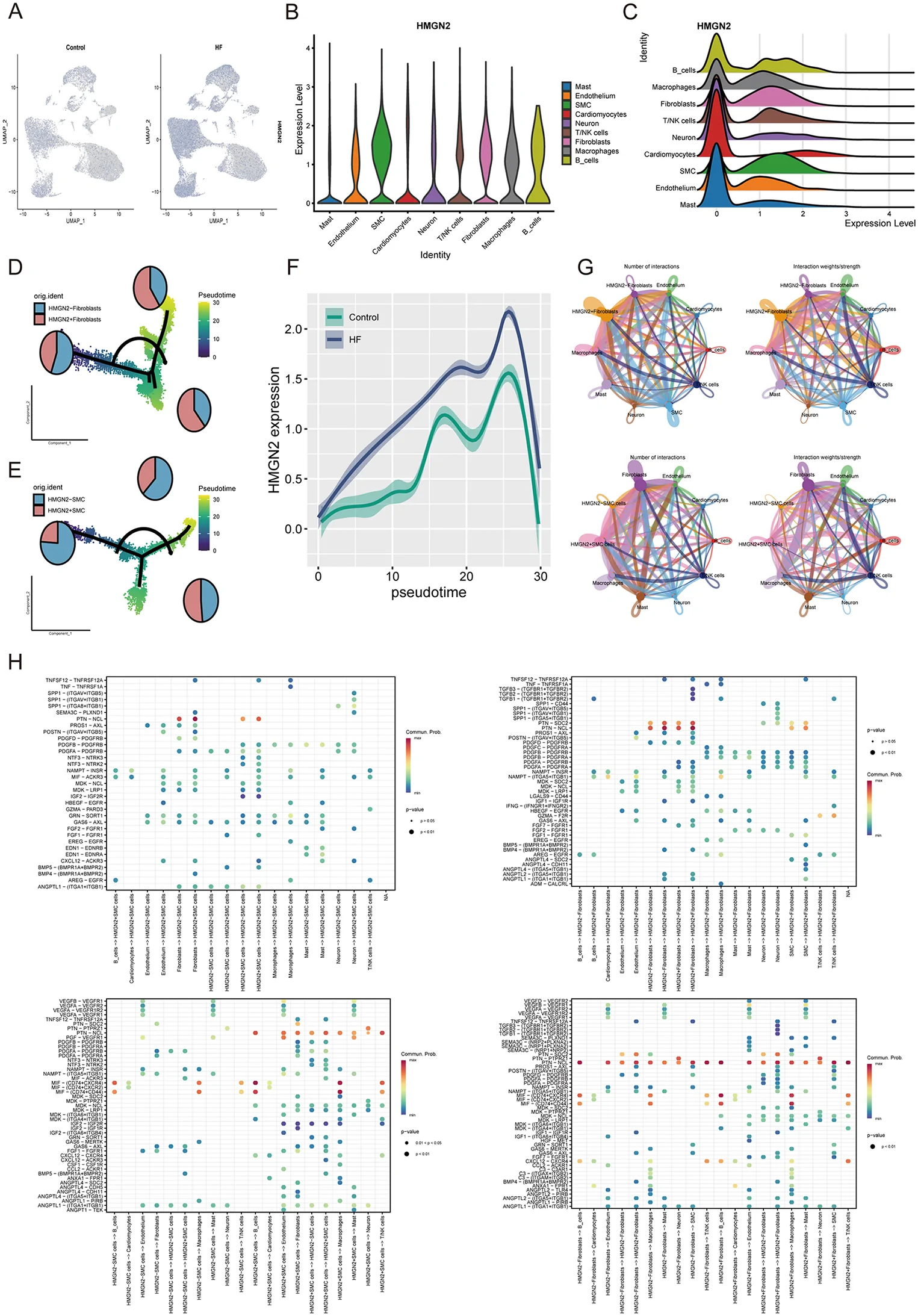
Single-cell heterogeneity of HMGN2 in HF. A. UMAP shows HMGN2 expression concentrated in fibroblasts and SMCs. B-C. Violin and density plots confirm high HMGN2 in fibroblasts/SMCs, low in mast cells and cardiomyocytes. D-F. Trajectory analysis reveals HMGN2 + fibroblasts and SMCs expand synchronously with divergent expression in HF. G. Altered cellular interactions between HMGN2 + / − cells and others. H. HMGN2 + cells communicate via specific ligand-receptor pairs (e.g., MIF-CD74/CXCR4, PTN-NCL).

To investigate single-cell expression heterogeneity of HMGN2 in the HF microenvironment, its distribution across cell types was analyzed using integrated UMAP dimensionality reduction, violin plots, and stacked density plots. UMAP clustering revealed that HMGN2 expression was predominantly concentrated within fibroblast and SMC populations (Fig 7A). In alignment, violin plots and ridge density analyses validated high levels of HMGN2 expression in fibroblasts/SMCs, in contrast to minimal expression in mast cells and cardiomyocytes (Fig 7B and 7C). Fibroblasts and SMCs were stratified into HMGN2 + / − Fibroblasts and HMGN2 + / − SMCs. Trajectory analysis was performed using Monocle2 to investigate transcriptional heterogeneity in fibroblasts and SMCs. During quasitemporal ordering, HMGN2 + fibroblasts and HMGN2 + SMCs exhibited synchronized proportional expansion. Pseudotemporal analysis revealed distinct relative expression patterns of HMGN2, with divergent trends along pseudotime between Control and HF groups (Fig 7D–7F). Cellular communication analysis revealed differences in both the number and strength of interactions between HMGN2 + /HMGN2 − SMCs, fibroblasts, and other cell types (Fig 7G). Compared to HMGN2 − fibroblasts, HMGN2 + fibroblasts exhibited stronger outgoing and incoming interaction strengths, suggesting their heightened participation in cellular communication. Similarly, HMGN2 + SMCs displayed higher activity in both efferent and afferent interactions compared to HMGN2 − SMCs (S2 Fig). Ligand-receptor interaction analysis further elucidated the connectivity between HMGN2 + /HMGN2 − SMCs, fibroblasts, and other cell types. HMGN2 + SMCs interacted with other cells through receptor-ligand pairs such as MIF- (CD74 + CXCR4), PTN-NCL, and PGF-PEGFR1. Similarly, HMGN2 + fibroblasts communicated via PTN-NCL, MIF- (CD74 + CD44), and CXCL12-CXCR4 pairs (Fig 7H). Heatmap analysis highlighted elevated communication probabilities in HMGN2 + fibroblasts/SMCs, alongside complex signaling networks involving diverse cell types. HMGN2 + fibroblasts exhibited heightened activity in pathways, including IGF, PROS, MK, and MIF, whereas incoming signals such as Periostin and TGF-β were predominantly expressed in HMGN2 − fibroblasts. Additionally, HMGN2 + SMCs displayed enhanced activity in ANGPT, CSF, and VEGF pathways, with incoming signals like PROS and PDGF preferentially expressed in HMGN2 + SMCs. These signaling molecules play crucial roles in regulating cellular behaviors, participating in tissue repair, ECM remodeling, and immune modulation (S3 Fig).

### 3.8 Experimental validation of optimal feature genes in H9C2 cells and TAC mouse model

**Fig 8:**
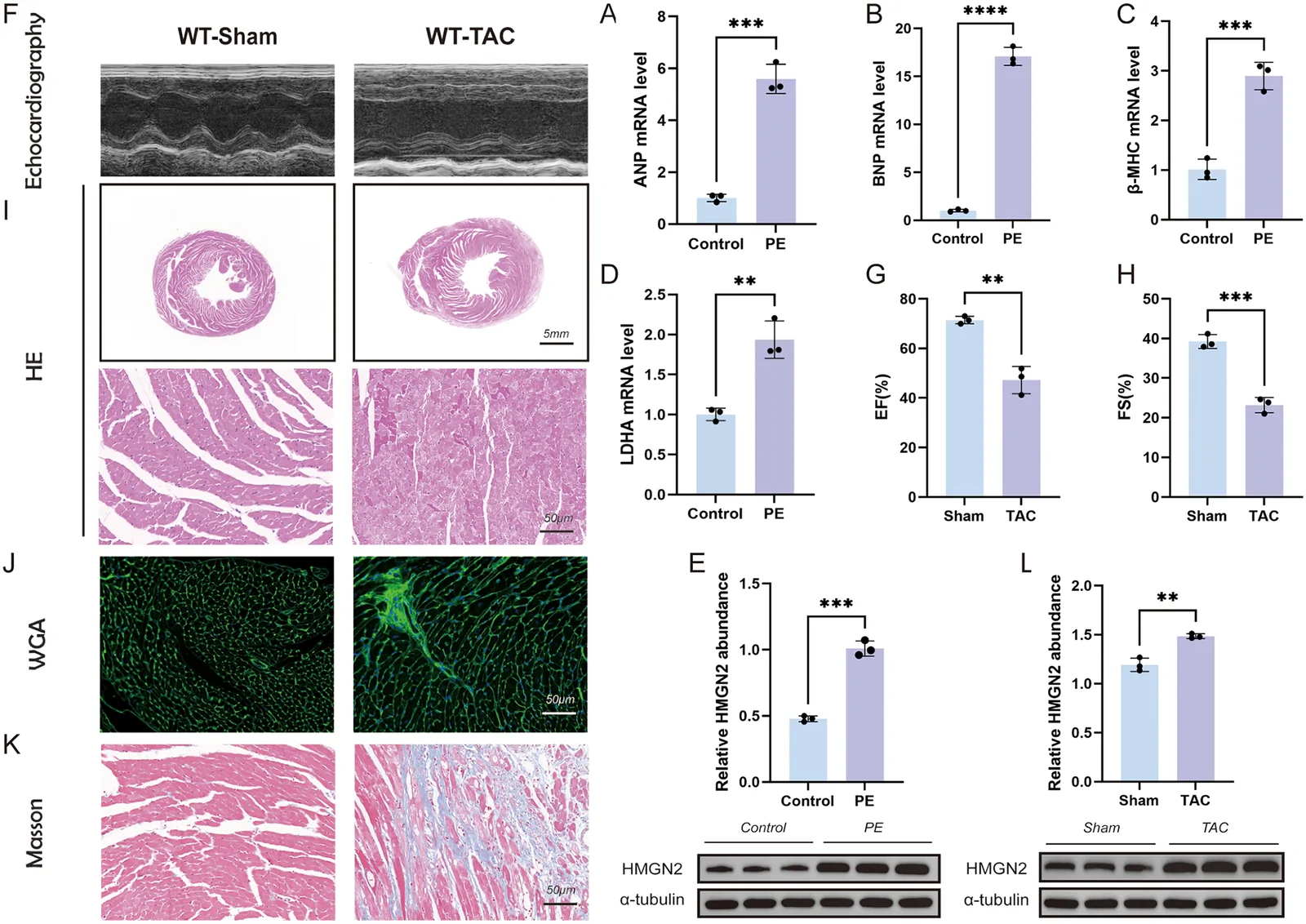
HMGN2-induced lactylation and cardiac dysfunction in cellular/animal HF models. A–C. PE-induced upregulation of ANP, BNP, and β-MHC mRNA in H9C2 cells confirms hypertrophic modeling. D. LDHA elevation indicates increased lactylation activity after PE treatment. E. HMGN2 protein is upregulated under PE stimulation. F–H. TAC operation impairs cardiac function, reducing EF% and FS%. I–K. Histology reveals hypertrophy, fibrosis, and structural disruption in TAC hearts. L. HMGN2 is consistently elevated in TAC mice. Data are presented as mean ± SEM. n = 3 independent biological replicates per group for all in vitro and in vivo experiments unless otherwise stated.

It is worth noting that while our scRNA-seq analysis primarily highlighted the involvement of fibroblasts and smooth muscle cells, primary cardiac fibroblasts are prone to spontaneous activation and phenotypic drift *in vitro*. Therefore, as an initial proof-of-concept, we utilized the stable H9c2 cardiomyocyte cell line for our preliminary *in vitro* validation.To elucidate the role of HMGN2 in HF pathogenesis, H9C2 cardiomyocytes were treated with PE (100 µM) and 0.1% Brdu in high-glucose basal medium for 48 h to simulate HF conditions, while control cells received Brdu alone. Total RNA was isolated, reverse-transcribed, and analyzed via quantitative PCR (qPCR). PE treatment significantly increased the mRNA expression of ANP, BNP, and β-MHC (Fig 8A–8C), confirming successful HF modeling. Moreover, LDHA mRNA was markedly upregulated, indicating elevated lactylation activity (Fig 8D). Western blotting demonstrated enhanced HMGN2 protein levels in PE-stimulated cells compared to controls (Fig 8E). To further assess the functional relevance of HMGN2 in vivo, a TAC-induced HF mouse model was utilized. Echocardiographic analyses revealed substantial impairment of cardiac contractility (Fig 8F), characterized by reduced EF (EF%) and FS (FS%) (Fig 8G and 8H). H&E, WGA, and Masson staining validated the presence of hypertrophy, fibrosis, and architectural disruption (Fig 8I–8K). Strikingly, HMGN2 expression was broadly elevated in TAC hearts (Fig 8L). Together, these results demonstrate that HMGN2 expression is upregulated concurrently with markers of hypertrophy and potential lactate metabolism (LDHA), supporting its positive association with HF progression, although further investigation is required to establish causal relationships.

## 4. Discussion

Our integrative scRNA-seq analysis reveals a marked elevation of lactylation signatures in the failing heart, particularly within SMCs and fibroblasts. The chromatin-binding protein HMGN2 emerges as the predominant hub gene driving these alterations. The lactylation score we computed was significantly higher in HF samples relative to controls, and HMGN2 expression showed a strong correlation with lactylation activity, suggesting its potential role in mediating pathological remodeling in HF.

Lactate, once considered merely metabolic waste, is now recognized as a versatile energy substrate. It is taken up via monocarboxylate transporter-1 (MCT1) and converted to pyruvate to meet energetic demands during hypoxia or exertion; yet excessive accumulation lowers intracellular pH and contributes to contractile dysfunction [1]. The discovery of histone lysine lactylation established a direct link between glycolysis and gene regulation: lactate-derived lactoyl-CoA can be transferred onto lysine residues by the acetyltransferase p300 and removed by HDAC1/2 [34]. Zhao et al. demonstrated that hypertrophic cardiomyocytes exhibit increased histone lactylation dependent on glycolysis, and that exogenous lactate enhances hypertrophic gene expression, whereas glycolysis inhibition reduces it [35]. These findings underscore that lactate not only fuels metabolism but also acts as an epigenetic signal. Furthermore, broader metabolite-host interactions, such as gut microbiota metabolic reprogramming, have also been shown to drive systemic disease development [36].

Our results extend previous work by placing lactylation within a cell‑type–specific context. Bioinformatic analyses have identified several LRGs in HF, and our machine‑learning screening demonstrates that HMGN2 surpasses these genes in predictive power. HMGN2 modulates chromatin structure by interacting with TFs such as Lef‑1, Dlx2, and Pitx2, and binding active chromatin marks, including H4K5ac and H3K4me2 [37]. We observed a strong correlation between HMGN2 expression and lactylation scores across fibroblast trajectories. Single‑gene gene‑set enrichment analysis linked HMGN2 to inflammatory pathways and ECM organization, consistent with studies showing that histone lactylation promotes reparative gene activation and regulates fibrosis [38,39].

Identifying fibroblasts and SMC as lactylation‑enriched clusters underscores the importance of cell‑type specificity. Cardiac fibroblasts are central to scar formation and fibrotic remodeling; myocyte loss leads to fibroblast activation and ECM deposition, and the adult heart has limited regenerative capacity [40]. Fibroblasts undergoing metabolic reprogramming export lactate through MCT4 and supply it to cardiomyocytes via MCT1, fueling hypertrophic growth [41]. Our observation of high lactylation in SMCs suggests that multiple stromal cell types participate in the lactate–lactylation axis. Trajectory analysis showed that lactylation scores and HMGN2 expression increase along pseudotime in fibroblasts and SMC, implying progressive epigenetic reprogramming during disease progression. These findings complement studies demonstrating that maintaining histone lactylation via METTL7B protects against HF [39] and that lactylation in macrophages drives reparative programs after myocardial infarction [38].

Our study also reconciles divergent reports on lactate and lactylation. Elevated serum lactate and lactate/albumin ratios are associated with increased mortality in HF patients [8], and chronic lactate accumulation may activate inflammatory pathways such as NLRP3 inflammasome–mediated pyroptosis [42]. In contrast, exogenous lactate supplementation improves cardiac function in acute HF: lactate infusion in porcine models of cardiogenic shock and small randomized trials increased cardiac output and stroke volume without adverse effects [43,44]. Similarly, lactate administration maintains histone lactylation and attenuates remodeling in hypertrophic cardiomyopathy models. These seemingly opposing outcomes likely reflect differences in timing and localization. Acute HF involves hypoperfusion and energetic deficit, where lactate serves as a substrate and lactylation activates reparative genes; chronic pressure overload results in persistent lactate production, intracellular acidification, and excessive lactylation of regulatory proteins, which may drive inflammation and fibrosis [45]. By demonstrating that HMGN2‑dependent lactylation is heightened in fibroblasts and SMC, our single‑cell data suggest that lactylation is maladaptive in established HF but potentially beneficial when transient or targeted.

Despite these advances, our study has several limitations. This analysis is cross‑sectional and based on limited single‑cell datasets, preventing us from delineating the temporal dynamics or causal relationships between lactylation and HF progression.

Future research should employ longitudinal sampling, spatial transcriptomics, and mechanistic experiments to elucidate how HMGN2 interacts with lactylation writers and erasers, and whether modulating HMGN2 expression can halt or reverse fibrotic remodeling. Furthermore, exploring pharmacological interventions targeting fibroblast activation offers promising translational potential. For instance, recent comprehensive reviews have highlighted the robust cardioprotective and anti-fibrotic effects of natural compounds, such as Motherwort, in mitigating myocardial remodeling and multi-organ injury [46]. Measuring circulating and intracellular lactate and lactoyl‑CoA alongside epigenomic profiling will clarify systemic versus cell‑intrinsic contributions. Stage‑specific interventions targeting lactate transporters MCT1 and MCT4, or enzymes controlling lactylation, could enhance histone lactylation during acute injury while preventing excessive lactylation during chronic overload. Integrating single‑cell epigenomics with metabolic and functional assays may ultimately translate the lactate–lactylation axis into targeted therapies for HF.

Several limitations of this study should be acknowledged. First, the 332 LRGs used to calculate the enrichment score were derived from an HCC context [11]. While the core lactylation machinery is biologically conserved, future studies should establish HF-specific lactylation gene sets for sensitivity validation. Additionally, our scRNA-seq analysis was based on a relatively small cohort (2 controls and 4 HF patients). While this sample size is consistent with exploratory single-cell studies and adequate for identifying major cellular shifts, it may lack the statistical power to detect subtle differences in rare cell subpopulations. Larger-scale scRNA-seq cohorts are required to further validate these findings.Second, our bioinformatic scoring and the *in vitro* detection of LDHA mRNA upregulation represent *inferred* lactylation activity and lactate production potential, respectively. These surrogate markers provide preliminary conceptual evidence but do not equate to direct measurements of histone or protein lactylation. Nevertheless, a recent landmark study has provided robust mechanistic validation for this axis: Chen et al. demonstrated that LDHA-dependent lactate production is the direct upstream driver of cardiac histone lactylation (e.g., H3K18la), and that targeted inhibition of LDHA effectively suppresses histone lactylation and alleviates HF progression [47]. Therefore, our LDHA findings align with established biochemical pathways, though future investigations utilizing mass spectrometry-based lactylome analysis (e.g., employing Pan-Kla antibodies) are still essential to directly quantify protein lactylation levels. Third, due to the retrospective nature of public transcriptomic datasets, comprehensive clinical metadata were unavailable. Consequently, we were unable to perform multivariate regression analyses to fully adjust for potential confounders such as age, sex, specific HF etiologies, and diverse treatment backgrounds. Furthermore, the TAC model in this study only utilized male mice to reduce estrogenic interference. Given the known sex-based differences in cardiovascular diseases, future studies incorporating both male and female groups are necessary to fully evaluate the sex-specific effects of HMGN2. Finally, although our single-cell analysis highlighted fibroblasts as a primary hub of inferred lactylation, our *in vitro* validation utilized the H9c2 cardiomyocyte cell line; future studies should utilize primary cardiac fibroblasts or conditional knockout mouse models to fully elucidate cell-specific mechanisms and avoid the spontaneous phenotypic drift often seen *in vitro*. Additionally, while HMGN2 demonstrated robust diagnostic performance independently within each dataset, the lack of longitudinal clinical cohorts limits our ability to evaluate its long-term prognostic value. Future prospective, longitudinal cohort studies are required to dynamically monitor HMGN2 expression during HF progression and further validate its clinical utility.

## 5. Conclusion

This work delivers a cell-type-resolved map of lactylation in HF, revealing that epigenetic reprogramming is a defining feature of the disease. Single-cell RNA-seq integration and five independent lactylation scoring methods demonstrate that lactylation activity is significantly enhanced in SMCs and fibroblasts, while neurons and cardiomyocytes remain relatively inert. Consensus machine learning analysis identified seven candidate genes; HMGN2, NUCKS1, and VIM were markedly up-regulated, with HMGN2 showing the highest diagnostic consistency across human cohorts and a murine model. Functional enrichment and TF-miRNA regulatory network analyses implicate HMGN2 in immune-inflammatory signaling, protein homeostasis, and potential fibroblast activation. Pseudotime trajectories and inferred cell-cell interactions indicate a progressive increase in both lactylation scores and HMGN2 expression along fibroblast and smooth muscle pathways, reflecting dynamic epigenetic reprogramming during disease evolution. Experimental validation in a pressure overload mouse model reproduced the hypertrophic and fibrotic phenotype and confirmed elevated HMGN2 expression.Collectively, these data underscore lactylation as a key factor associated with HF and highlight HMGN2 as a candidate therapeutic target for arresting pathological cardiac remodeling.

## Supporting information

S1 Fig Results of enrichment analysis. Gene Ontology and KEGG pathway enrichment analysis for the identified differentially expressed genes or gene clusters in heart failure.
(TIF)

S2 Fig The interaction dynamics among different cell types. Cellular communication network inferred from single-cell RNA-seq data, demonstrating the interaction strength and number of interactions across distinct cell populations.
(TIF)

S3 Fig Results of signaling pattern heatmap. Heatmap displaying the major outgoing and incoming signaling pathways for each identified cell type in the study.
(TIF)

S1 Raw Images Original uncropped western blot membranes. This file contains the original, uncropped, and unadjusted blot images for Fig 8.
(ZIP)

S1 Data Original data analysis. This file contains the raw data and quantitative analysis results for qPCR and histological experiments.
(ZIP)

S2 Data Dataset and analysis code. This archive contains the relevant datasets and custom code used for the bioinformatic and machine learning analyses in this study.
(ZIP)
